# Commentary: Association between life’s essential 8 and testosterone deficiency in US men: findings from national health and nutrition examination survey (NHANES) 2011–2016

**DOI:** 10.3389/fendo.2025.1492712

**Published:** 2025-02-20

**Authors:** Linkun Shen, Sheng Li, Fei Zhang

**Affiliations:** ^1^ Department of Urology Surgery, Ningbo No.2 Hospital, Ningbo, Zhejiang, China; ^2^ Department of Anorectal Surgery, Ningbo No.2 Hospital, Ningbo, Zhejiang, China

**Keywords:** life’s essential 8, testosterone deficiency, NHANES, cardiovascular health, cross sectional study

## Introduction

We read with the article by Mei et al. entitled “Association between life’s essential 8 and testosterone deficiency in US men: findings from the national health and nutrition examination survey (NHANES) 2011–2016” (1), which explores the association between “Life’s Essential 8” (LE8) and testosterone deficiency (TD) among adult men in the United States. The research was based on data from the National Health and Nutrition Examination Survey (NHANES) 2011–2016, analyzing 4,971 men aged 20 and older according to the inclusion criteria set by the authors. Cardiovascular health was scored according to the American Heart Association’s (AHA) LE8 criteria. Authors constructed three weighted multivariate logistic regression models to explore the relationship between LE8 scores and testosterone deficiency. Model 1 provided crude odds ratios and did not adjust for any covariates. Model 2 adjusted for age, race, marital status, and education level. Model 3 further adjusted for BMI, smoking, alcohol consumption, hypertension, and diabetes. Subgroup analyses were conducted based on age, BMI, and smoking status to examine the association between LE8 and testosterone deficiency in different subgroups. They concluded that there was a significant negative association between LE8 scores and testosterone deficiency. For every 10-point increase in LE8, the risk of testosterone deficiency decreased by 21% (OR=0.79). Notably, the association between health factors (such as BMI and blood glucose) and testosterone deficiency was more pronounced, whereas the relationship between health behaviors (such as diet and exercise) and testosterone deficiency was weaker. This study innovatively explores the relationship between LE8 scores and testosterone deficiency, proposing that LE8 could be applied in clinical practice to help identify the risk of testosterone deficiency in men at an early stage. While we greatly appreciate the authors’ efforts, we still have some concerns regarding the study.

## Covariate adjustment

The authors included demographic covariates such as age, race, marital status, education level, and PIR in their models, which we highly commend. However, we noticed that in Model 3, the authors adjusted for smoking status, alcohol intake, hypertension, diabetes, cardiovascular disease, and hyperlipidemia. According to the AHA’s definition of LE8, the LE8 score includes eight elements: diet quality, physical activity, smoking status, sleep duration, BMI, blood lipids, blood glucose, and blood pressure. This suggests that changes in these covariates could affect the outcome of the exposure variable. The authors did not mention whether a collinearity analysis was conducted, and we believe that the adjustment for smoking status, hypertension, diabetes, cardiovascular disease, and hyperlipidemia in Model 3 may have influenced the study’s conclusions.

## Outcome variable selection

In this study, the authors designated testosterone deficiency as the outcome variable, defining it as a single serum testosterone level <300 ng/dL, and used this as the sole inclusion criterion. However, research by Brambilla et al. (2) pointed out that hormones with pulsatile secretion, such as testosterone, have substantial short-term variability. Among men whose initial testosterone levels were in the hypogonadal range (<300 ng/dL), approximately 30% had normal testosterone levels on repeat measurement. In community-based cohorts of middle-aged and older men of different races, daily variations in serum testosterone levels were so significant that a single testosterone measurement was insufficient to represent an individual’s testosterone levels (3). Therefore, according to the guidelines of the American Urological Association (4), at least two testosterone measurements are needed to confidently diagnose TD. This limitation could affect the overall findings of the study.

## Discussion

In summary, although we appreciate the valuable contribution of this study to our understanding of the relationship between LE8 and testosterone deficiency, we believe that addressing these issues and considering other factors would enhance the robustness and comprehensiveness of the study’s results. We look forward to the authors addressing these concerns to further improve this research.
